# Exploring Protein Misfolding in Amyotrophic Lateral Sclerosis: Structural and Functional Insights

**DOI:** 10.3390/biomedicines13051146

**Published:** 2025-05-09

**Authors:** Ouliana Ivantsik, Themis P. Exarchos, Aristidis G. Vrahatis, Panagiotis Vlamos, Marios G. Krokidis

**Affiliations:** 1 Bioinformatics and Human Electrophysiology Laboratory, Department of Informatics, Ionian University, 49100 Corfu, Greece; 2 Institute of Digital Biomedicine, University Center for Research and Innovation, Ionian University, 49100 Corfu, Greece

**Keywords:** amyotrophic lateral sclerosis, protein misfolding, aggregation, prion, mutations, superoxide dismutase 1

## Abstract

Protein functionality depends on its proper folding, making protein misfolding crucial for the function of proteins and, by extension, cells and the whole organism. Increasing evidence supports the role of protein misfolding in the pathogenesis of neurodegenerative diseases, such as amyotrophic lateral sclerosis (ALS). ALS is a rapidly progressive disease diagnosed at a prevalence of 5 cases per 100,000, with approximately 2–3 patients per 100,000 diagnosed each year. To date, there is no cure, and the disease usually leads to death within 2 to 5 years from diagnosis. There are two types of the disorder: familial ALS (fALS), accounting for approximately 10% of cases, and sporadic (sALS), accounting for the remaining 90%. The hallmark of ALS, regardless of type, is the protein aggregates found in patients’ tissues. This suggests that the disruption of proteostasis plays a critical role in the development of the disease. Herein, we stress the distinct factors that lead to protein misfolding and aggregate formation in ALS. Specifically, we highlight several triggering factors affecting protein misfolding, namely mutations, errors in the processes of protein production and trafficking, and failures of folding and chaperone machinery. Gaining a deeper understanding of protein aggregation will improve our comprehension of disease pathogenesis and potentially uncover new therapeutic approaches.

## 1. Introduction

For efficient cellular function, a variety of functional proteins is required. The proper functioning of these proteins depends on their correct three-dimensional structure [1]. Under normal conditions, proteins naturally fold into the most energetically stable form while the way they fold is influenced by the primary sequence of the polypeptide and the environmental conditions [2]. In the absence of triggering factors that affect protein folding, the process is completed successfully, and a functional protein is produced. Sometimes, however, folding can occur incorrectly, leading to the production of misfolded proteins, which may have no function or functions different from the wild type [3]. Factors that can affect the protein folding procedure include mutations in the gene sequence, errors in the processes of transcription or translation, failures of the folding and chaperone machinery, mistakes in the post-translational modifications or trafficking of proteins, structural modifications produced by environmental factors, the induction of protein misfolding by seeding and cross-seeding mechanisms, pH changes, infections, and aging [4]. During protein misfolding, aberrant protein assemblies can be produced that favor the aggregation of these proteins, an event associated with the pathology of many diseases [5].

Protein misfolding is a key factor in a variety of diseases, including the most common neurological diseases, such as Alzheimer’ s disease (AD), Parkinson’ s disease (PD), amyotrophic lateral sclerosis (ALS), frontotemporal dementia (FTD), Huntington’s disease (HD), and prion diseases [6], as well as some rare inherited disorders that involve the aggregation of protein misfolding and promote harmful conditions, such as amyloidosis [7]. Misfolded proteins lead to the pathogenesis and progression of diseases through two mechanisms: The first involves the accumulation of misfolded proteins inside or outside the cell, resulting in the disruption of the normal function of the cell and the underlying tissue. A typical example is the extracellular accumulation of Aβ and the intracellular accumulation of p-tau proteins in the brains of AD patients [8]. The second pathological property of misfolded proteins is the prion-like mechanism, resulting in their dissemination to different anatomical structures of the nervous system, leading to the progression of the disease [9].

ALS is a progressive neurodegenerative disease that leads to paralysis and subsequent death in a very short period, usually 2 to 5 years from diagnosis. The prevalence of the disease is estimated to be 5 cases per 100,000, with approximately 2–3 patients per 100,000 diagnosed each year, figures that demonstrate the rapid progression of the disease to death. Survival depends on several factors, with bulbar onset, delay in diagnosis, and the presence of cognitive impairment and specific mutations being associated with a worse prognosis [10]. The disease can be sporadic (sALS), accounting for approximately 90% of cases, or familial (fALS). Unfortunately, to date, there are no treatments that delay the progression of the disease or significantly increase patient survival, highlighting the importance of a better understanding of the pathological mechanisms for identifying appropriate therapeutic strategies. Regardless of the type of disease or the clinical picture, the common feature observed in post-mortem tissue is protein inclusions (Bunina bodies, basophilic inclusions, skein-like inclusions, and hyaline inclusions) in nerve cells and adjacent tissues [11]. The location of the inclusions, as well as their composition, varies significantly from patient to patient. Approximately 97% of ALS cases exhibit TDP-43-positive inclusions. The presence of these protein inclusions indicates that the main pathogenetic mechanism for the onset of the disease is the disruption of protein homeostasis in the nervous system [7].

As mentioned previously, the folding of the polypeptide depends on its sequence and on the environmental conditions. The main proteins involved in the onset of ALS are C9orf72, SOD1 (superoxide dismutase 1), TDP-43, and FUS (fused in sarcoma). For all of the above proteins, there is evidence that their accumulation can lead to the occurrence of ALS [11], but it is interesting that aggregates of these proteins are observed, even in the absence of mutations, indicating that further mechanisms leading to protein misfolding are involved in the pathogenesis of ALS. Thus, the purpose of this review is to discuss the mechanisms involved in protein misfolding and to correlate them with data from ALS research, shedding light on the possible triggering factors of the disease. Understanding these causes is critical for developing prevention and treatment strategies for ALS.

## 2. Genetic Mutations in ALS

The three-dimensional shape of a protein is determined by the amino acid sequence. Thus, the most common cause of protein misfolding is genetic mutations that affect the amino acid sequence. The formation of these structures ensures that hydrophobic regions are enclosed in the interior of the structure, so that they do not come into contact with the aqueous environment of the cell. Misfolding effects result from mutations that cause the incorrect exposure of parts of the polypeptide that should be inside the protein to the protein surface. Thus, these regions produce a highly adhesive protein surface, leading to the consequent production of aggregates [3]. Many mutations have been implicated in the occurrence of ALS. Approximately 70% of fALS and 15% of sALS are due to mutations in more than 50 known genes, with *SOD1*, *FUS*, *TARDBP*, and *C9ORF72* being the most frequently mutated genes. In fact, several of the mutations identified in these genes are suggested to lead to pathogenesis through disrupting the protein folding process [12]. In this section, we discuss the mutations that lead to protein aggregation of the SOD1, FUS, and TDP-43, the pathological inclusions of which are typically found in ALS patients. Table 1 summarizes the mutations in each gene and the mechanisms through which some of them are identified to contribute to further protein misfolding in the nerve cell.

Today, more than 185 mutations have been identified in the *SOD1* gene, most of which are missense [12]. It was initially proposed that mutations caused a decrease in the activity of the dismutase enzyme, but subsequent studies showed that the activity of the enzyme was not related to the severity of the disease [13], indicating that the mutations likely led to the onset of the disease not through loss of function, but through toxic gain. Specifically, it is proposed that the mutations that lead to the pathogenesis of ALS are those that affect the structural stability of the protein through the collapse of the homodimeric complex, resulting in aggregation [14]. Of the mutations found, 10 have been shown to be associated with the protein misfolding of SOD1: A4V, W32S, G37R, L38V, H46R, G85R, D90A, G93A, G93C, and G127X (Table 1).

TDP-43 inclusions are found in ~97% of ALS patients [15]. These aggregates are caused by proteins that are mislocalized in the cytoplasm and are abnormally phosphorylated, proteolytically processed, and ubiquitinated. At least 48 mutations in the gene have been identified to date, the majority of which are nonsynonymous. A total of 22 mutations have been shown to be associated with the protein misfolding of TDP-43 in nerve cells (Table 1). One mutation is located in the nuclear localization signal (A90V), one in an RNA recognition motif (D169G), seventeen in the C-terminal low-complexity domain (G290A, G294A, G294V, G295C, G298S, A315T, A315E, A321G, Q331K, M337V, Q343R, N345K, G348C, N352S, G358C, R361S, S379C, A382T, and N390D), and one in a non-functional region (K263E). Of particular interest, however, is the fact that pathological mutations in TDP-43 are observed in only 1–2% of ALS patients [15], and there are no reports that specific mutations cause TDP-43 protein misfolding. This indicates that other mechanisms cause misfolding and the aggregation of TDP-43.

FUS-related ALS is characterized by the pathological aggregation of the FUS protein and has only been observed in patients carrying pathological variants in the *FUS* gene. More than 50 mutations associated with ALS have been identified in this gene, the majority of which are missense mutations [12]. Many of these are located in the nuclear localization signal, while others affect glycine and arginine-rich regions, the prion-like domain, and the 3′ UTR [16]. Several of the mutations that have been recorded affect protein folding, altering the ability of the protein to form solid aggregates, thus leading to the pathogenesis of ALS [17]. Specifically, 10 mutations are identified that cause protein misfolding. One is located in the Gln/Gly/Ser/Tyr-rich region (G156E), which is a prion-like domain, and one in an arginine/glycine-rich region (R495X). The rest of mutations are located in the proline/tyrosine–nuclear localization signals (R514G, R521C, R521H, R521G, R522G, R524S, P525L, and P525R). Another mechanism of pathogenesis involves the inhibition of the nuclear transport of FUS, resulting in cytoplasmic aggregation. The most frequently affected regions are the amino acids 510–526 [16]. Mutations in this region disrupt the interaction between the proline–tyrosine nuclear localization signal (PY-NLS) of FUS and its nuclear import receptor (transportin-1). In fact, a direct correlation has been found between how mutations in *FUS* affect the affinity for transportin-1 and the onset and progression of the disease [18].

**Table 1:** SOD1, TDP-43, FUS, and C9orf72 mutations and the protein aggregation trigger mechanism that contributes to protein folding.

Protein	Mutation	Induction of Further Protein Misfolding	Reference
SOD1	A4V	Accumulation of ubiquitins into inclusions Disruption of Golgi trafficking Endoplasmic reticulum stress	[14,19,20]
	W32S	Prion-like seeding	[21]
	G37R	N/A	[22]
	L38V	N/A	[14]
	H46R	Prion-like seeding	[23]
	G85R	Prion-like seeding Disruption of Golgi trafficking	[20,24]
	D90A	N/A	[25]
	G93A	Disruption of autophagy Disruption of axonal transport Disruption of Golgi trafficking Accumulation of ubiquitins into inclusions Induction of Golgi fragmentation Prion-like seeding	[26,27,28]
	G93C	N/A	[14]
	G127X	Prion-like seeding	[24]
TDP-43	A90V	Prion-like seeding	[29]
	D169G	N/A	[30]
	K263E	N/A	[31]
	G290A	Affects the proteasome	[32]
	G294A	Prion-like seeding	[33]
	G294V	Affects the proteasome	[32]
	G295C	N/A	[34]
	G298S	N/A	[31,35]
	A315T	Errors in protein synthesis Affects the proteasome	[32,36]
	A315E	Prion-like seeding	[37]
	A321G	N/A	[38]
	Q331K	N/A	[39]
	M337V	Accumulation of ubiquitins into inclusions Affects the proteasome	[19,32]
	Q343R	Errors in protein synthesis	[40]
	N345K	N/A	[39]
	G348C	Stress induction	[32,41]
	N352S	Affects the proteasome	[42]
	G358C	N/A	[34]
	R361S	N/A	[39]
	S379C	N/A	[34]
	A382T	Affects the proteasome	[32]
	N390D	N/A	[39]
FUS	G156E	Prion-like seeding	[43]
	R495X	Accumulation of ubiquitins into inclusions	[19,44]
	R514G	N/A	[45]
	R521C	Errors in protein synthesis SMN incorporation	[46]
	R521H	Errors in protein synthesis Inclusion of SMN	[46]
	R521G	Errors in protein synthesis	[47]
	R522G	N/A	[47]
	R524S	N/A	[44]
	P525L	SMN incorporation Errors in protein synthesis Disruption of autophagy	[17,46,48]
	P525R	Errors in protein synthesis	[49]
C9orf72 (gene)	CpG methylation	Errors in protein synthesis Disruption of nucleocytoplasmic transport	[50,51]

N/A; not applicable.

## 3. Errors in the Protein Synthesis Processes

Errors in protein synthesis, i.e., transcription and translation, have been repeatedly associated with the development of neurodegenerative diseases and especially ALS. In fact, specific mutations in proteins involved in these processes seem to trigger the misfolding and aggregation of the generated proteins, resulting in their accumulation inside or outside the cell [48]. Concisely, evidence supports that the aggregation of RNA-binding proteins inhibits the transport of RNA along neuronal microtubules, consequently suppressing localized translation within dendrites and potentially resulting in aberrant translation in the cellular environment [52].

A proteomic analysis of FUS aggregates indicated the dysregulation of proteins involved in the translation process [53]. The authors also showed that key translation proteins co-localize with FUS inclusions in neural cells carrying *FUS* mutations and observed the negative regulation of translation in patient-derived fibroblasts. This is consistent with the results of another study, which documented reduced protein synthesis in the growth cones of cultured *Xenopus* retinal ganglion neurons in cells expressing mutant FUS [54]. However, it seems that FUS aggregates do not only affect translation through a reduction in translation. Imaging data supports that the presence of FUS inclusions causes ectopic protein expression, leading to an imbalance in the local protein production between the neuronal periphery and the cell soma [55]. Also, several FUS mutations seem to cause aggregates that affect the translation process, causing errors (R521C, R521H, R521G, P525L, and P525R) (Table 1). A feature of FUS and TDP-43 aggregates is that they can incorporate other proteins from the organism, thus reducing their available reserve. A typical example is the adenomatous polyposis coli (APC) protein, which has been found to be incorporated into FUS inclusions in the cell cultures of patient-derived cells carrying *FUS* mutations [55]. Another case is the ribonucleoprotein (RNP) assembly protein survival motor neuron (SMN), which is also frequently entrapped in FUS R521C, R521H, and P525L aggregates. SMN plays a role in splicing and small nuclear ribonucleoprotein (snRNP) biogenesis, while also participating in the transport of RNAs into axons and in local translation [56].

The protein TDP-43 plays a key role in the transcription and processing of mRNA transcripts, with data supporting that it has more than 6000 target transcripts (approximately one-third of the transcriptome) [15]. In the case of an increased TDP-43 concentration, binding to RACK1 of polyribosomes has been observed, leading to an influence on translation while axonal TDP-43 condensates inhibit the local protein synthesis of nuclear-encoded mitochondrial proteins that are important for neuromuscular junctions [57]. Specifically, a study proved that mutant TDP-43 A315T and Q343R cause errors in protein synthesis [40]. Intrinsically disordered, aggregation-prone domains at the C-terminal region of hnRNP A2/B1 and hnRNP A1, as well as TDP-43, have been observed, suggesting their fibrillization and misfolding [58]. Τhe hnRNP A1 isoform, whose upregulation is induced by depletion, has been reported to be highly sensitive to TDP-43 depletion [59]. C9ORF72 also appears to affect translation. Translation of this gene results in the production of dipeptides poly(GA), poly(GP), poly(GR), poly(PA), and poly(PR), which have been identified in patient tissues. In the context of ALS, these dipeptides are proposed to affect translation and nucleocytoplasmic transport. The proposed model is that the hydrophobic dipeptides produced by the translation of *C9ORF72* inhibit translation by preventing the binding of translation factors to mRNA [50].

## 4. Errors in Protein Trafficking

Vesicular transport-related proteins, such as OPTN, VAPB, CHMP2B, and UNC13A, have been associated with ALS, suggesting that faulty vesicular transport contributes to the pathophysiology of the disease [60]. According to several studies, prolonged inhibition of vesicular trafficking from the Golgi to the plasma membrane can result in protein accumulation and consequently Golgi fragmentation, common characteristics of ALS patients’ neurons [61]. Evidence suggests that Golgi apparatus fragmentation may be a cause of neurodegeneration rather than a result of it, occurring early in the pathological cascade of disease.

**Figure 1:**
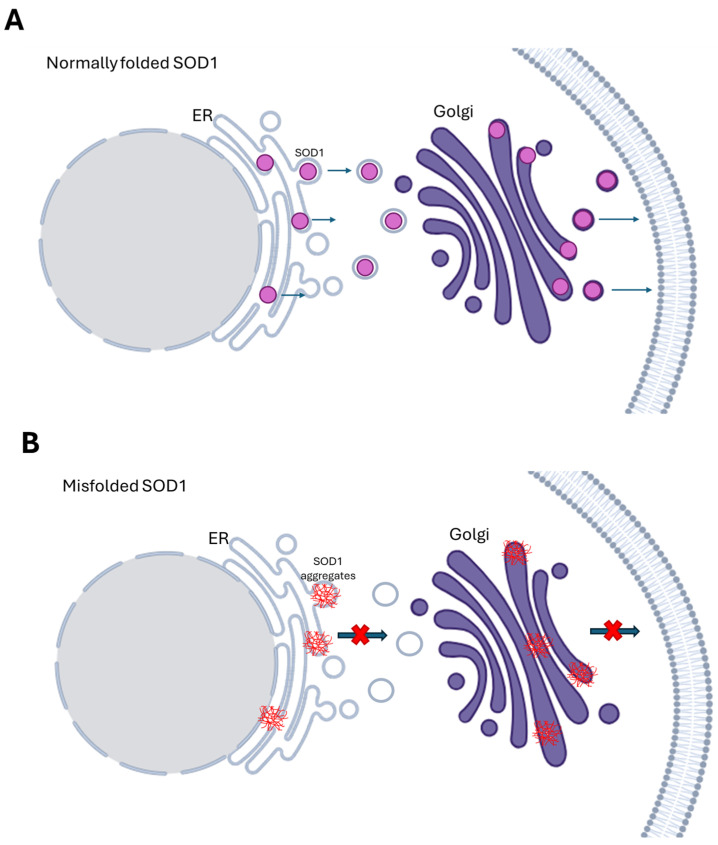
SOD1 protein trafficking at the Golgi apparatus. (**A**) Normally folded SOD1 and protein transport from the ER to the Golgi. (**B**) Different mutations in SOD1 affect protein trafficking at the Golgi in different ways. The ALS-associated mutations SOD1A4V, SOD1G85R, and SOD1G93A disrupt the secretory pathway, while the SOD1A4V mutation triggers the accumulation of secretory proteins and apoptosis.

Specifically, mutations in *SOD1* have been found to affect trafficking at the Golgi through two mechanisms: the ALS-associated mutations SOD1A4V, SOD1G85R, and SOD1G93A disrupt the secretory pathway, while the SOD1A4V mutation triggers endoplasmic reticulum (ER) stress causing the accumulation of secretory proteins and apoptosis [20], as presented in Figure 1. The SOD1-G93A is also found to induce Golgi fragmentation and disrupt axonal transport (Table 1). Additionally, mutations in TDP-43 and FUS may disrupt axonal transport by causing stress granule formation and protein aggregation or by decreasing the availability of neurofilaments [62]. FUS mutant proteins’ aggregates sequester SMN proteins, reducing their availability. These proteins are crucial for mRNA trafficking in the axon [63]. Similarly, TDP-43 aggregates in ALS patients contain many nuclear import and export proteins and mutant TDP-43 disrupts the nuclear membrane and nuclear pore complex (NPC), resulting in the decreased import of nuclear proteins and export of RNA [64]. The ALS-associated protein aggregates that develop because of compromised nucleocytoplasmic trafficking may further affect the nucleocytoplasmic transport of proteins and RNAs [65]. Finally, it has been suggested that repeat-expanded *C9ORF72* also influences cytoplasmic and nuclear trafficking. Affected motor neurons and other brain and spinal cord cells accumulate transcripts of *C9ORF72* that include expanded repeats. According to several studies, these stable hexanucleotide repeat-containing *C9ORF72* RNA species sequester components of the NPC, including RanGAP1 and RNA-binding proteins. This disrupts their and other proteins’ nucleocytoplasmic trafficking and function [66].

## 5. Dysfunction of the Folding and Chaperone Machinery

**Figure 2:**
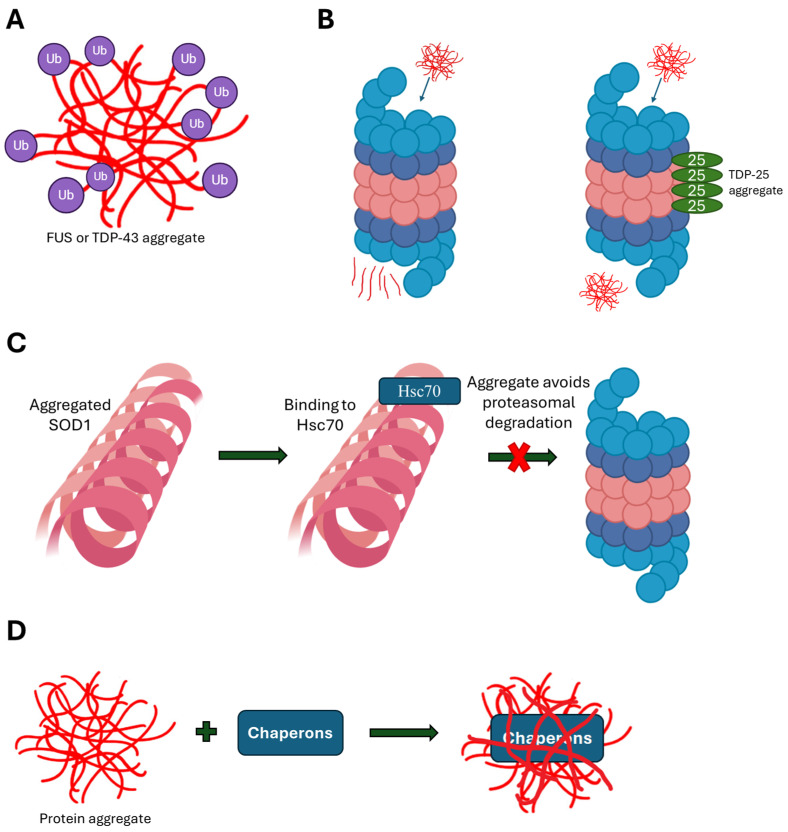
The mechanisms through which ALS-associated aggregates impact protein folding machinery. (**A**) Mutants TDP-43 and FUS tend to aggregate and undergo ubiquitination, leading to a significant reduction in free ubiquitins. (**B**) TDP-25 binds proteasome subunits, preventing them from undergoing the conformational changes necessary for enzymatic activity. (**C**) SOD1 aggregates incorporate Hsc70, which protects SOD1 aggregates from proteasomal degradation. (**D**) ALS-linked mutant protein aggregates often bind and integrate chaperones, diminishing their active potential.

Foldases and chaperones are responsible for the correct folding of proteins, preventing the toxic effects and aggregation of misfolded proteins. Specifically, they inhibit aggregation, refold, cooperate with proteases to facilitate protein degradation, and disaggregate protein aggregates [67]. Protein misfolding and aggregation are increasingly associated with the occurrence of neurodegenerative diseases [6]. The main mechanism of the pathogenesis of ALS is the accumulation of protein aggregates, with most patients carrying TDP-43 aggregates. Mutants TDP-43-M337V and FUS-R495X tend to aggregate and undergo ubiquitination, leading to a significant reduction in free ubiquitins (Figure 2A) [19]. Another mechanism is that TDP-25, as a fragment of TDP-43 consisting of the aggregated TDP-43 mutants G290A, G294V, A315T, M337V, N352S, or A382T, binds to proteasome subunits, preventing them from undergoing the conformational changes necessary for enzymatic activity [34], as Figure 2B indicates.

Mutant aggregates of SOD1 also appear to function by a similar mechanism. The intraneuronal aggregates in some ALS patients with mutants SOD1-A4V and G93A contain ubiquitinated SOD1 [68], and in many cases Hsc70 is found in these aggregates [69]. Binding to Hsc70 protects SOD1 aggregates from proteasomal degradation, favoring their accumulation (Figure 2C) [69]. Furthermore, chaperones responsible for dealing with ALS-linked mutant protein aggregates often bind and integrate into them, diminishing their active potential (Figure 2D) [70]. Studies have shown significant levels of various chaperones, among them HSP70and VCP; co-chaperones such as BAG3, HSP40, and calreticulin; and protein folders such as HSP110 (HSPH family), proSAAS (proprotein convertase subtilisin/kexin type 1 inhibitor, a granin-like protein), and MIF (migration inhibitory factor) in SOD1, C9orf72, FUS, and TDP-43 aggregates [71,72].

## 6. Seeding and Cross-Seeding Mechanisms

Following the initial formation of misfolded proteins, the aggregations could impact the progress of the folding of other normally folded proteins, leading to their misfolding and consequent accumulation, a mechanism known as “seeding and cross-seeding” [73]. Thus, in the first stage, the lag phase, thermodynamically unfavorable protein structures (due to misfolding) are formed until the smallest stable oligomeric unit is created. These function as seeds for the next stage, the elongation phase, where the polymers grow quickly. In vitro studies have demonstrated this mechanism for SOD1, where adding recombinant SOD1 aggregates to cultured cells demonstrated SOD1’s capacity to misfold and/or aggregate in a prion-like way in living cells [23]. Another study showed that endogenous wild-type SOD1 might become misfolded when ALS-associated mutant SOD1 is overexpressed in cultured cells [24]. This mechanism was subsequently shown to operate not only within the same cell, but also intercellularly. Two primary pathways of spread have been proposed: the packing of misfolded protein seed into or on extracellular vesicles known as exosomes, and the release of aggregates from dying cells [74]. A further study of SOD1 revealed that there are certain sequence regions that favor the formation of amyloid fibrils. Based on 3D profile approaches, these were found to cause the induction of the amino acid sequence segments _14_VQGIINFE_21_, _30_KVWGSIKGL_38_, _101_DSVISLS_107_, and _147_GVIGIAQ_153_ [75]. Moreover, there are several mutations that have been identified to induce prion-like aggregate propagation: W32S, H46R, G85R, G93A, and G127X, as Table 1 indicates.

Similar findings have been observed for TDP-43. It has been shown that upon the lipofection of the C-terminal hemagglutinin-tagged TDP-43, the protein aggregates in the cell cytoplasm, forming polyubiquitinated and sarkosyl insoluble cellular aggregates [76]. Another study performed lipofection of the insoluble protein fraction of human sALS or TDP-43-positive FTD patient brain samples in a neuronal cell [77]. The authors noticed that when the FTD or sALS samples were added to cells, HA-tagged positive TDP-43 inclusions formed. Then, the inclusions created in cultivated cells were used to seed more aggregation in the cultures of naïve cells, making the results even more robust. Subsequent research using stable cultures [78], overexpression cultures [79], and even the axonal uptake and transport of TDP-43 seeds [80] revealed comparable results for the cell-to-cell spread of aggregation. Further investigation was carried out to indicate which regions of the protein are responsible for this mechanism. Since the low-complexity domain (LCD) is considered to be a prion-like domain (PrLD) when focusing on its physicochemical properties, which consist of asparagine, glutamine, tyrosine, and glycine residues, this was the first region suspected as being responsible for the aggregation propensity of TDP-43 [81]. Three-dimensional profiling methods and crystallography experiments revealed that six segments of the LCD region could form steric zippers, a structural motif of amyloid fibrils: _300_GNNQGSN_306_, _321_AMMAAA_326_, _328_AALQSS_333_, _333_SWGMMGMLASQ_343_, _370_GNNSYS_375_, and _396_GFNGGFG_402_ [82]. In addition to the LCD, other steric zipper polymorphs have been proven to form from a segment of RRM2 (RNA Recognition Motif 2) (_247_DLIIKGISVHI_257_) [83], indicating that this domain may also play a role in pathological aggregation, at least when RRM2 is partially folded [84]. The specific mutations found that lead to prion-like propagation are as follows: A90V, G294A, and A315E (Table 1).

The N-terminal QSGY-rich domain (residues 1–165) of FUS is also considered as a PrLD that may contribute to the abnormal aggregation of FUS seen in ALS patients [85]. Purified FUS PrLD has been examined and found to be capable of undergoing a phase transition to create hydrogels made of fibrils that resemble amyloid [86]. Furthermore, the G156E mutation seems to favor the transition from liquid to fibrous solid FUS and these fibrils may seed pure wild-type FUS [87]. Table 2 summarizes the main mechanisms leading to ALS pathogenesis through disrupting the protein folding process.

## 7. Aging

The average age of diagnosis of ALS is 55 years, with aging being one of the main risk factors for the disease. Aging is defined as the gradual, ongoing loss of an organism’s ability to function normally throughout life. It is also characterized by a higher probability of death and an increased susceptibility to diseases associated with aging. According to the World Health Organization (WHO), the percentage of people over 60 is projected to almost double (from 12% to 22%) between 2015 and 2050, suggesting that the prevalence of age-related neurodegenerative diseases such as ALS will increase sharply in the coming decades [96]. According to the literature, two main mechanisms have been reported through which aging leads to the occurrence of ALS, due to the promotion of protein misfolding: proteostasis loss and macrophage dysregulation.

Proteostasis collapse is a key feature of human cellular aging. During aging, the expression of protein chaperones, like the heat shock proteins (HSPs), declines, meaning that protein folding deteriorates. In fact, several studies show that the restoration of proteins whose expression is lost in cellular or animal models leads to increased life expectancy, delayed aging, and improved aging-related characteristics, such as cognitive skills [97]. Another mechanism affected in aging and which is related to proteostasis is that of stress granules (SGs) [98]. The production of SGs is controlled by liquid phase separation (LLPS), the mechanism by which proteins and nucleic acids in solution separate into liquid droplets. Several misfolded proteins linked to ALS, including TDP-43 and FUS, have domains that induce LLPS [99]. SGs also preserve proteostasis by sequestering misfolded proteins and preventing their accumulation in the cytoplasm or nucleus. Proteostasis is disrupted by aberrant SGs, and the loss of proteostasis is linked to problems in normal aging that regulate the normal assembly/disassembly and dynamics of SGs [100].

Dysregulation of macroautophagy has also been well documented in aging. Age-related decreases in the expression of genes linked to autophagy, such as ATG5, ATG7, and OPTN, have been observed [101]. As a result, as people age, protein clumps and malfunctioning organelles build up. Furthermore, studies have shown that autophagy stimulation or activation lengthens lifespans and improves health in both humans and animal species. ALS-associated mutations in *C9ORF72*, *SOD1* (G93A), *TARDBP*, *TBK1*, *FUS* (P525L), *FIG4*, *OPTN*, *UBLN2*, *SQSTM1*, *CHMP2B*, and *ALS2* dysregulate macroautophagy and dysregulated macroautophagy is linked to neurodegeneration in ALS [101,102]. It should be noted that in ALS, the pharmacological or genetic inhibition of autophagy accelerates aging and increases motor neuron toxicity, while autophagy is essential for the removal of protein aggregates associated with ALS neurodegeneration.

## 8. Other Factors

### 8.1. pH Changes

Experimental studies have shown that pH is a factor that affects protein folding and unfolding, and pH changes have been implicated in several neurological diseases [103]. pH can affect the protein folding process directly through changing the thermodynamic stability of tertiary structures, as well as indirectly affecting the mechanisms involved in protein folding and aggregation. These include the changing activity of proteases; the relocation of acidified organelles, such as lysosomes to the peripheral zones of cells; the fusion of lysosomes with redistributed compartments that contain amyloid precursor protein (APP); and the disruption of chaperone function through promoting conformational changes in peptidyl-prolyl cis-trans-isomerase (Pin 1 or PPIase), an endogenous chaperone, leading to reduced chaperone activity [103].

In ALS, an acidic environment has been implicated in the disruption of several cellular processes, such as the reduction in glutamate reuptake, the mitochondrial vacuolization, the glial cell activation, the endoplasmic reticulum stress, and the impairment of oxidative phosphorylation/ATP synthesis. Also, acidosis triggers the activation of acid-sensing ion channels (ASICs), which, when overstimulated, lead to intracellular Ca^2+^ influx, neuroinflammation, axonal degeneration, demyelination, and neuronal death [104]. The effects of cellular pH on TDP-43 localization, aggregation, and phosphorylation have been studied recently on a cellular model [105]. Although no changes were observed in TDP-43 expression, mislocation of the protein from the nucleus to the cytoplasm was observed, which can be attributed to a change in the conformation of the protein that does not allow its correct transport to the nucleus.

### 8.2. Environmental Factors

As mentioned above, ALS has a familial and a sporadic form, suggesting that environmental factors probably play a major role in the pathogenesis of the disease. The environmental factors that have been associated with ALS pathogenesis are heavy metals, pesticides, β-N-methylamino-L-alanine (BMAA), and physical activity (professional football in particular) [106]. Investigating the mechanisms through which these factors may lead to ALS pathogenesis, protein misfolding is found to be the mechanism responsible for the pathogenesis due to heavy metals and BMAA. Experimental studies suggest that BMAA leads to neurotoxic effects through several mechanisms, including its ability to induce protein misfolding, and the accumulation of intracellular aggregates [107].

**Figure 3:**
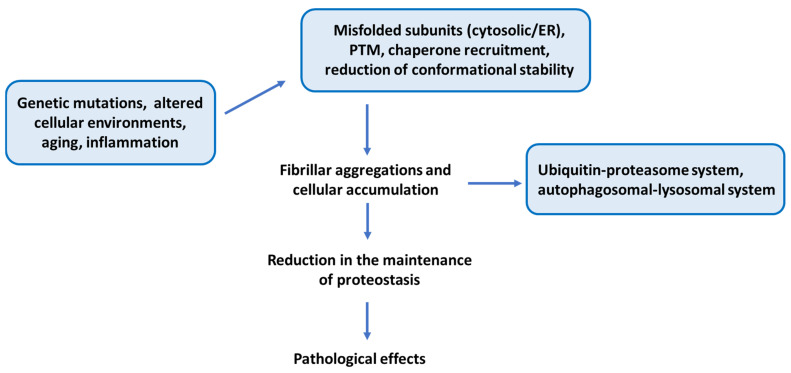
A summarized figure that recapitulates the main points of the present work emphasizing protein misfolding, aggregation, and dysfunctions in ALS.

There are several mechanisms through which heavy metals can cause protein misfolding. Firstly, they can directly affect the protein by replacing essential metal ions (like zinc and copper) that are crucial for the proper folding and stability of proteins. This substitution can lead to structural changes in proteins, leading to misfolding [108]. Also, heavy metals can bind to specific sites on proteins, altering their conformation. Indirectly, heavy metals disrupt cellular processes that are essential for protein folding, interacting with chaperone proteins, protein synthesis, and degradation. Finally, heavy metals can generate reactive oxygen species (ROS), leading to oxidative stress [109]. This oxidative environment can damage proteins, resulting in misfolding and aggregation. Research on the hypothesis that heavy metals can contribute to the etiology of ALS raises controversial results. A recent systematic review investigated the association between human tissue lead levels and ALS and the meta-analysis concluded that ALS patients have higher lead levels than controls in blood (standardized mean difference (SMD) = 0.61; 95% confidence interval (CI)—0.20, 1.01; *p* = 0.003), plasma/serum (SMD = 0.27; 95% CI—0.16, 0.70; *p* = 0.26), and cerebrospinal fluid (CSF) (SMD = 0.53; 95% CI—0.09, 1.15; *p* = 0.09), suggesting an association between lead tissue bioaccumulation and ALS [110]. Figure 3 summarizes the involved processes which are described in the present work, such as protein misfolding, aggregation, and dysfunctions in ALS.

## 9. Structural Aspects of ALS-Related Proteins

SOD1 is a 32 kDa homodimeric metalloenzyme, in which the two subunits composed of 153 amino acids in the human isoform are linked with a disulfide bridge. Each subunit is formed by a β-barrel core and seven loops. Loops IV and VII are extremely important from structural and functional perspectives as six out of the seven metal co-ordination sites take place into these two loops [111]. The metal binding site co-ordinates a Cu and a Zn ion at the active site by loop IV that facilitate SOD1’s functionality in its catalytic activity by binding one copper and one zinc ion per monomer and stabilize its conformation by the disulfide bond between Cys57 in loop IV and Cys146 in β-strand β8. The human copper chaperone for SOD1 (hCCS) transiently associates with monomeric SOD1, promoting metal incorporation and disulfide bond formation between Cys57 and Cys146 within the subunit, ultimately resulting in the generation of dimeric holo-SOD1 [112]. The misfolded species constitutes the precursor to SOD1 aggregation, which may arise through the exposure of hydrophobic surfaces, localized unfolding of the central β-barrel, polymerization mediated by extended active site loops, oxidative crosslinking between Cys6-Cys111, or an integration of these mechanisms [113].

TDP-43 is a 414 amino acid heterogeneous ribonucleoprotein with a modular structure comprising several distinct domains. It comprises two conserved RNA-recognition motifs (RRMs) followed by a glycine-rich C-terminal domain; an N-terminal domain containing a nuclear localization signal and a nuclear export signal; and a C-terminal prion-like domain [114]. Intrinsically disordered regions are a significant feature of TDP-43, providing challenges for the structural determination of the full-length protein. Pathological TDP-43 aggregates are composed of the full-length protein and of abnormally cleaved C-terminal fragments (CTFs) of 18–35 kDa [115]. The low-complexity domain comprises the glycine-rich region, which is disordered and essential for protein–protein interactions, such as binding to heterogeneous nuclear ribonucleoproteins, whereas the A315T, Q331K, and M337V mutations promote protein aggregation [116].

The full-length human FUS protein contains 526 amino acids. The N-terminal proximal half of FUS contains a QGSY-rich region and a Gly-rich domain, while its C-terminal proximal half includes two Arg/Gly-rich domains intervened by a Zn finger, an RNA recognition motif, and a C-terminal Pro/Tyr-rich region comprising a nuclear localization signal [117]. The C-terminal half of the FUS protein incorporates two Arg-Gly-Gly repeat regions interrupted by a cysteine–cysteine zinc finger motif, an RNA recognition motif, and a non-conventional nuclear localization signal interacting with the nuclear transport receptor Transportin 1 [93].

### Post-Translational Modifications

Post-translational modifications (PTMs) have been observed in various amino acids of SOD1, including lysine, tryptophan, and serine. For example, lysine is subjected to mutations (K4E) and can be modified through glycation and acetylation, Trp33 fulfills the roles of both an oxidation site and a nitration site, while Ser60 serves as a phosphorylation site [118]. Zinc binding and an internal disulfide bond help form the SOD1 homodimer interface, supported by the terminal regions and inter-subunit hydrogen bonds. Copper addition boosts the protein’s stability and enzymatic activity [119]. Without these post-translational modifications (PTMs) and dimer formations, SOD1 becomes unstable, unfolded, and prone to aggregation. Notably, engineering a disulfide bond in the A4V mutant reduced aggregation and restored function, indicating dimer stabilization as a potential therapeutic approach [119].

Phosphorylation and ubiquitination are the two main pathological PTMs in TDP-43. The phosphorylation of TDP-43 within TDP-43-positive inclusions in brain samples has been thoroughly characterized, as a result of the availability of highly specific antibodies capable of identifying site-specific phosphorylation positions. The TDP-43’s ubiquitination is currently being extensively examined [34]. It has also been observed that the acetylation of specific lysines in C-terminal fragments of TDP-43 reduces the aggregation capacity. On the other hand, the spontaneously formed cysteine-linked homodimers found in recombinant TDP-432 C seem to elevate the amyloidogenicity [120]. Furthermore, the acetylation of Lys510 in the NLS promotes the formation of FUS-containing cytoplasmic aggregates while acetylation at Lys315/Lys316 within the RNA recognition motif domain of FUS substantially modifies the ability of binding RNA [121].

## 10. Conclusions

Protein aggregates are the main characteristic of ALS, both in familial and sporadic form. This highlights the connection between protein misfolding and ALS pathogenesis. The present review aims to delineate the complex association underlying the two occurrences, highlighting the multiple mechanisms through which distinct factors lead to the pathogenesis of ALS mediated by protein misfolding events. It appears that various factors enhance protein misfolding in the disease, strengthening the hypothesis that ALS is a multi-factorial disease; however, further research is required to examine these processes more thoroughly. Emphasis should be given to the promotion of the accumulation of misfolded proteins through a deficiency in the autophagy mechanism. The dynamic process of autophagy includes the formation of an autophagosome which then fuses with lysosomes and degrades. Through this process, the accumulation of autophagosomes occurs and promotes viral growth by providing membrane scaffolds for viral assembly and replication. Also, the disruption of the autophagy process leads to the accumulation of protein aggregates, resulting in pathological manifestations [102]. Limitations include the destabilization of the wild-type SOD and its activity uber desaturating conditions as well as the strong disulfide environment of the cytosolic compartment which is highly resistant to proteolysis. Although considerable progress has been made in elucidating the disease, the precise processes through which proteins misfold, aggregate, and lead to neurodegeneration are still not fully understood. Furthermore, despite evidence that the synaptic environment may promote protein misfolding due to its supersaturated state, the origin of this process—whether synaptic or somatic—remains unclear.

The present study also carries noteworthy implications for the prevention and treatment of this incurable disease. Numerous promising therapeutic strategies targeting SOD1, TDP-43, or aggregated proteins have been highlighted, including interaction modulators or small molecules as well as the modulation of protein degradation pathways or the use of antisense technologies. The implication of the enhanced expression of Hsps should be highlighted, as this participates in the clearance of aggregated proteins, autophagy-mediated removal of misfolded proteins, and removal of protein aggregations by the upregulation of proteasomal degradation mechanisms [122]. Given their essential roles in maintaining protein homeostasis—from translation to degradation—as well as mediating cellular responses to stress, Hsps represent a compelling, though challenging, therapeutic target for protein misfolding disorders such as ALS. It would be interesting to further explore how therapeutic interventions targeting these mechanisms could interrupt the cycle of protein misfolding and aggregation to halt disease progression as well as deplete already-formed aggregates to repair pre-existing damage.

## Figures and Tables

**Table 2:** Mechanisms through which SOD1, FUS, TDP-43, and C9ORF72 lead to ALS pathogenesis through disrupting the protein folding process.

Protein	Process Affected	Mechanism	References
SOD1	Mutations in the gene sequence	Collapse of the homodimeric complex, resulting in aggregation	[88]
	Errors in the protein trafficking	ALS-associated mutations SOD1A4V, SOD1G85R, and SOD1G93A disrupt the secretory pathway	[20]
		SOD1A4V mutation triggers ER stress causing the accumulation of secretory proteins and apoptosis	[20]
	Dysfunction of the folding and chaperone machinery	SOD1 aggregates incorporate Hsc70, which protects the aggregates from proteasomal degradation	[89]
		SOD1 aggregates chaperones, minimizing their availability	[70]
	Seeding and cross-seeding mechanisms	ALS-associated mutant SOD1 causes wild-type SOD1 misfolding	[74]
TDP-43	Errors in the processes of protein production	TDP-43 binds to the RACK1 of polyribosomes, inhibiting protein synthesis	[90]
		Axonal TDP-43 condensates inhibit local protein synthesis	[57]
		TDP-43 depletion leads to the upregulation of specific isoforms of hnRNP A1, one of which has been reported to be highly prone to aggregation	[59]
	Errors in the protein trafficking	TDP-43 aggregates in ALS patients incorporate nuclear import and export proteins	[91]
		Mutant TDP-43 causes stress granule formation and protein aggregation	[62]
		Mutant TDP-43 disrupts the nuclear membrane and NPC	[64]
	Dysfunction of the folding and chaperone machinery	Mutant TDP-43 tends to aggregate and undergo ubiquitination, leading to a significant reduction in free ubiquitins	[19]
		The TDP-43 aggregation-prone prion-like domain binds to proteasome subunits, minimizing their availability	[32]
		TDP-43 aggregates chaperones, minimizing their availability	[70]
	Seeding and cross-seeding mechanisms	TDP-43 aggregates cause the aggregation of other proteins through seeding	[92]
FUS	Mutations in the gene sequence	Mutations affect protein folding, altering the ability of the protein to form solid aggregates	[17]
		Mutations inhibit the nuclear transport of FUS, resulting in cytoplasmic aggregation	[93]
	Errors in the processes of protein production	FUS inclusions incorporate key translation proteins	[53]
		FUS inclusions cause ectopic protein expression	[55]
		FUS inclusions incorporate the APC protein, affecting functions in nerve cells	[94]
		FUS inclusions incorporate the SMN protein that plays a role in protein translation	[46]
	Errors in the protein trafficking	FUS inclusions incorporate the SMN protein that plays a role in mRNA trafficking in the axon	[46]
		Mutant FUS causes stress granule formation and protein aggregation	[62]
		Mutant TDP-43 tends to aggregate and undergo ubiquitination, leading to a significant reduction in free ubiquitins	[19]
	Dysfunction of the folding and chaperone machinery	FUS aggregates chaperones, minimizing their availability	[70]
	Seeding and cross-seeding mechanisms	The G156E mutation favors the transition from liquid to fibrous solid FUS, and these fibrils seed pure wild-type FUS	[87]
C9ORF72	Errors in the processes of protein production	Dipeptides produced by *C9ORF72* translation inhibit translation by preventing the binding of translation factors to mRNA	[95]
	Errors in the protein trafficking	Hexanucleotide repeat-containing *C9ORF72* RNA species sequester components of the NPC, including RanGAP1 and RNA-binding proteins, disrupting their and other proteins’ nucleocytoplasmic trafficking	[66]
	Dysfunction of the folding and chaperone machinery	C9ORF72 aggregates chaperones, minimizing their availability	[70]

## Data Availability

No new data were created or analyzed in this study.
